# Internal bracing with suture tape augmentation reduces positive postoperative pivot shift in patients with anterior cruciate ligament reconstructions

**DOI:** 10.1002/jeo2.70200

**Published:** 2025-04-03

**Authors:** Simone Elmholt, Torsten Nielsen, Anders Galaly, Mogens Strange, Kaspar Saxtrup, Martin Lind

**Affiliations:** ^1^ Department of Othopaedic Surgery Aarhus University Hospital Aarhus N Denmark; ^2^ Department of Orthopedic Surgery Copenhagen University Hospital – Rigshospitalet København Ø Denmark; ^3^ Department of Sports Traumatology Silkeborg Regional Hospital Silkeborg Denmark; ^4^ Department of Orthopedic Surgery Mølholm Privthospital Vejle Denmark

**Keywords:** anterior cruciate ligament reconstruction, orthopedic surgery, sports traumatology, suture tape

## Abstract

**Purpose:**

In biomechanical testing synthetic ligament augmentation demonstrated improved graft strength, which may lead to improved clinical outcomes. Therefore, this study aimed to investigate whether this procedure would improve clinical outcomes, compared to conventional anterior cruciate ligament reconstruction (ACLR).

**Methods:**

This study was a retrospective register‐based cohort study. Data were obtained from a single clinic (Silkeborg Regional Hospital, Denmark). A cohort of patients undergoing ACLR with either hamstring or quadriceps tendon autografts in combination with an InternalBrace was identified. Using propensity scoring, the InternalBrace group was matched 1:1 to a control group of ACLR patients without augmentation from the same clinic. The primary outcome was sagittal knee laxity, and secondary outcomes were rotational stability (pivot shift), patient‐reported outcome measures (PROMs) with the Knee Injury and Osteoarthritis Outcome Score (KOOS) and revision surgery rates.

**Results:**

A total of 324 patients were included, 162 in each group. At 1‐year follow‐up the InternalBrace group demonstrated similar sagittal knee laxity of 2.2 mm (95% CI: 1.9–2.4 mm) compared to 1.9 mm in the control group (95% CI: 1.6–2.1 mm) (*p* > 0.05). The InternalBrace group demonstrated a statistically significant lower risk of having a positive pivot shift at the 1‐year follow‐up: 18% versus 30% (*p* < 0.01). There were no differences between the groups in the KOOS subcategories (*p* > 0.05 for all comparisons). At 2‐year follow‐up, two patients had a revision surgery in the InternalBrace group compared to three patients in the control group (*p* = 0.3).

**Conclusion:**

Compared to conventional ACLR, InternalBrace reduces the risk of having a positive pivot shift 1 year postoperatively and demonstrates similar outcomes regarding sagittal knee laxity, revision rates and PROMS. Therefore, this study concludes that InternalBrace are safe to use in ACLR.

**Level of Evidence:**

Level III.

**Abbreviations**
ACL: anterior cruciate ligament
ACLR: anterior cruciate ligament reconstruction
ADL: activity of daily life
DKRR: Danish Knee Reconstruction Registry
KOOS: Knee Injury and Osteoarthritis Outcome Score
PROMs: patient reported outcome measures
QOL: quality of life
QT: quadriceps tendon autografts
SD: standard deviation
SMD: standardised mean difference
SSD: side‐to‐side difference

## BACKGROUND

In recent years, there has been increasing interest in the use of synthetic ligament augmentation during anterior cruciate ligament reconstruction (ACLR) [9]. A synthetic ligament augmentation provides potential internal bracing of the ligament, acting as a “seatbelt” for the ACLR. Internal bracing of the anterior cruciate ligament (ACL) graft can be achieved using suture tape—an artificial suture composed of a non‐absorbable polyethylene/polyester—which is incorporated into the graft to strengthen the construct. This may protect the graft from excessive load during rehabilitation and return to sport [4, 21]. Especially during the postoperative healing phase, overloading of the graft construct may lead to soft‐tissue damage [2] and thus, impaired ACLR outcomes.

Biomechanical testing has shown that suture tape strengthens the graft construct by reducing graft elongation during cyclic loading and increasing the ultimate load [2, 16, 17, 22]. Only a few clinical studies have investigated whether tape augmentation can also result in clinically significant improvements compared with standard ACLR [1, 5, 15, 18, 24]. Most of these studies found no improvements in clinical outcomes with suture tape augmentation. In ACLR, revision surgery is considered the ultimate indicator of failure, and cohort studies comparing suture tape augmented hamstring grafts with hamstring grafts without augmentation found no difference in the graft rupture rates [15, 18, 24]. However, most of these studies were conducted on relatively small patient populations and were thus susceptible to being underpowered. A study of a larger patient cohort would add to the existing knowledge on the effect of suture tape on revision rates.

The aim of this study was to compare the clinical results of ACLR performed with and without suture augmentation using either a hamstring tendon autograft or a quadriceps tendon autograft. The primary outcome was sagittal knee laxity using either the KT‐1000 arthrometer or the Rolimeter. The null hypothesis was that there would be no difference in sagittal knee laxity between the groups. Rotational stability, patient reported outcome measures (PROMs) and revisions rates were included as secondary outcomes. For the secondary outcomes the null hypothesis was that there would be no difference between suture tape augmented ACLR and conventional ACLR.

## MATERIALS AND METHODS

### Study setting and data source

This comparative study retrospectively included data from a single clinic (Silkeborg Regional Hospital, Denmark) with two surgeons. The treatment and control groups were identified using the Danish Knee Reconstruction Registry (DKRR). DKRR is a large national database containing preoperative, operative and clinical outcomes of all ACLR procedures since 2005 [19]. The database contains data on ACLRs performed with synthetic ligament augmentation since 2019. This provides a unique opportunity to compare objective knee stability and revision surgery rates as well as subjective patient outcomes between ACLR with and without suture tape augmentation in a large patient population using prospectively collected data [7]. In the DKRR, all hospitals and surgeons have a unique code that ensures their identification in the registry.

The control group was formed using data from 2015–2017 from the same clinic. During this time period, the surgeons performed ACLRs without suture tape. From 2019 onwards, the surgeons have used suture tape augmentation for ACLR. Patients who had underwent primary ACLR with suture tape were included from 2019 to July 2021. Data were extracted from the DKRR on 1 July 2022 to ensure at least 1‐year of follow‐up for all included patients.

Preoperative, operative and postoperative data were collected from the registry. Preoperative data included age, sex, activity of injury, side of injury, previous surgery on the index knee, objective knee stability and PROMs. Information on the type of graft, cartilage injury, femoral drilling technique and meniscal injury and treatment was recoded from the surgery. One‐year follow‐up data included objective knee stability and PROMs. The date of the patient's primary ACLR and revision ACLR, if any, were also recorded.

This study was approved by the Danish Board of Health and the Danish Data Protection Agency (approval no.: 1‐16‐02‐216‐22). Since this study was based on data from a national healthcare registry, approval from the local ethical committee was not necessary.

### Study population

All patients who underwent ACLR during the specified study period were identified from the registry. Inclusion criteria were all ACLR performed in the specified clinic between 2015–2017 and 2019–July 2021 using the anteromedial portal femoral drilling technique and either hamstring or quadriceps tendon grafts. Exclusion criteria were other ligament injuries (i.e., posterior cruciate ligament, medial collateral ligament or lateral collateral ligament) and ACLRs performed with the transtibial drilling technique.

### Exposure

This study investigated the clinical outcomes of ACLR performed with suture tape augmentation (Arthrex Fiber Tape 2.0 mm) and either hamstring or quadriceps tendon autograft (QT). For hamstring autografts, the two strands of Fiber Tape were incorporated into the ACL graft by wrapping the tendons around the Fiber Tape. For the quadriceps tendon autografts, the strands of Fiber Tape were placed parallel to the ACL graft so that they ran along the graft inside the knee. All patients had undergone surgical reconstruction using the anteromedial portal femoral drilling technique. None of the patients underwent additional procedures such as anterolateral tenodesis.

### Outcomes

The primary outcome was sagittal knee laxity, assessed as the side‐to‐side difference (SSD) between the operated knee and the healthy knee, measured manually at 25° of flexion using either the KT‐1000 arthrometer or the Rolimeter [8]. Both preoperative and postoperative measurements were taken by an independent physiotherapist, using either the KT‐1000 arthrometer or the Rolimeter, depending on their preference. Objective knee stability was obtained in all patients who attended the 1‐year follow‐up. Secondary outcomes were rotational stability, PROMS and revision rates. The rate of revision surgery was assessed at both the 1‐year and 2‐year follow‐up as a cumulative rate. Rotatory knee instability was assessed with the pivot shift by an independent physiotherapist. The results were divided either according to no instability (pivot shift grade 0) or instability (pivot shift grade 1–3). The grading used for the pivot shift test was based on the IKDC grading system: Grade 0: normal, Grade 1: glide, Grade 2: clunk and Grade 3: locked subluxation. Subjective PROMS were evaluated by the Knee Injury and Osteoarthritis Outcome Score (KOOS) [20].

### Sample size calculation

The sample size was calculated using the following parameters and assumptions. In the total ACLR population, the SSD in sagittal laxity was 1.5 mm with a standard deviation (SD) of 1.5 mm. An increase in laxity of 1.0 mm would be considered clinically relevant. With a power of 0.8 and an alpha value of 0.05, a minimum of 63 patients per study group would be required.

### Sensitivity analyses

Baseline variables for patients excluded during the matching process were compared with those of the cohort population to assess for potential selection bias. As the suture augmentation group had undergone ACLR with both hamstring and quadriceps tendon autografts, a subgroup analysis was performed to assess whether there were differences in sagittal knee laxity between the two graft types. To assess whether the revision rates from this study were comparable to those in a national cohort, the treatment group was matched 1:1 using nearest neighbour matching to a new control group of patients from the national registry.

### Statistical analysis

To control for selection bias and confounding factors, the cohorts were matched using the propensity score [3, 14]. A propensity score was calculated for each patient using logistic regression with the following independent variables: age, sex, meniscal injury, cartilage injury, previous surgery in the index knee, type of injury (pivoting vs non‐pivoting) and preoperative pivot shift score (Grade 0 or Grade 1–3). By using the propensity score, the cohorts were matched 1:1 to obtain two comparable cohorts. Matching was performed using nearest neighbour matching with a caliper of 0.1. Baseline variables were compared using standardised mean difference (SMD), with SMD < 0.1 representing balanced variables.

Continuous parametric outcomes were analysed using the Student's *t*‐test, while non‐parametric data were analysed using the Wilcoxon rank‐sum test. Categorical outcome data were analysed using the Chi‐squared test. Follow‐up time from date of injury to surgery was calculated as the median. The rate of revision surgery at 1‐ and 2‐years was calculated using a hazard ratio. All analyses were conducted in STATA (ver. 18).

## RESULTS

**Figure 1:**
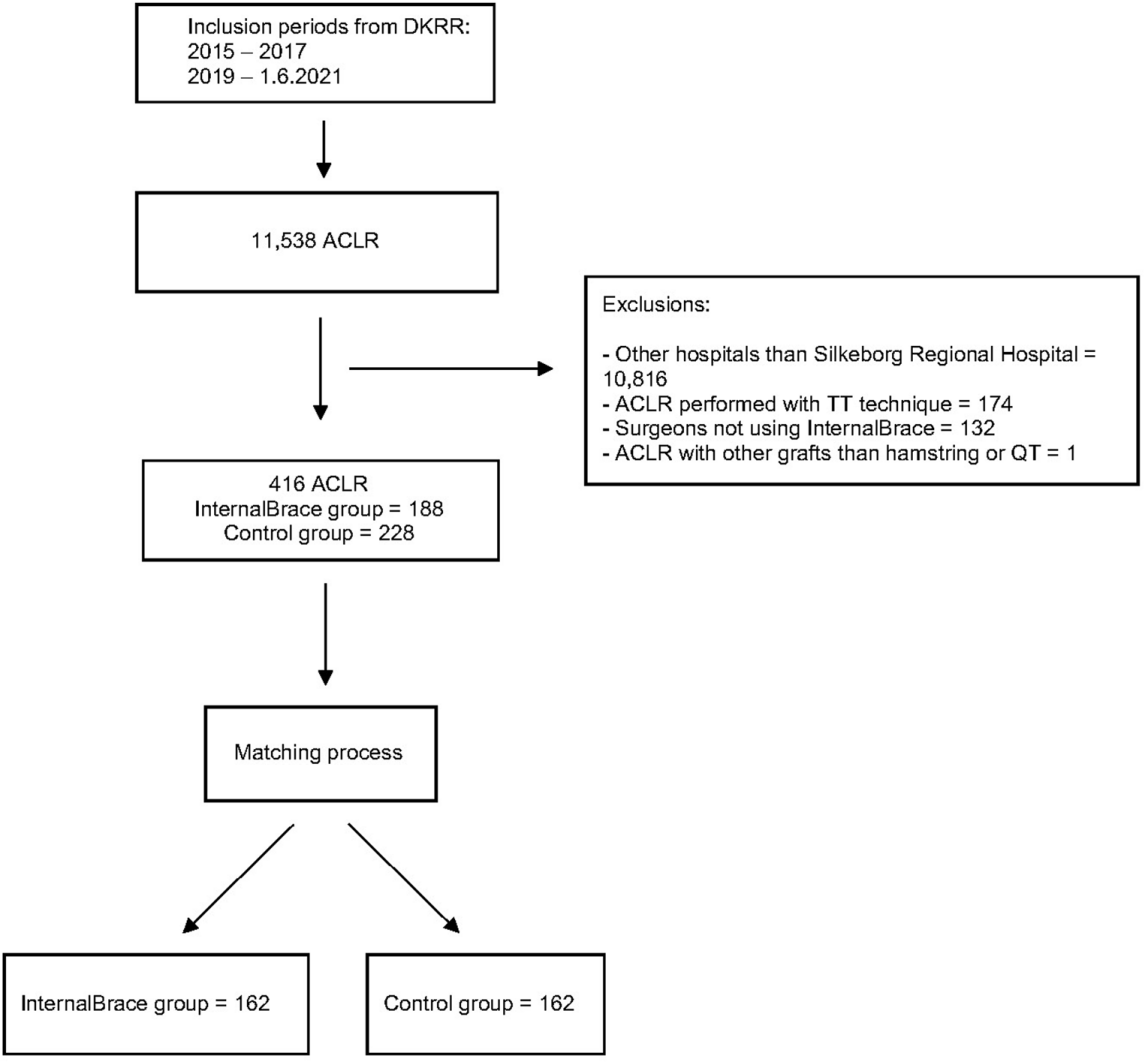
Flowchart demonstrating the inclusion and matching process. ACLR, anterior cruciate ligament reconstruction; DKRR, Danish Knee Reconstruction Registry; QT, quadriceps tendon autografts.

After exclusion, the treatment group consisted of a total of 188 patients who had undergone ACLR with suture augmentation. The control group consisted of 228 patients who had undergone ACLR without suture augmentation. After matching, both the treatment and control groups consisted of 162 patients, giving a total of 324 patients included in the study (Figure 1).

Baseline variables are shown in Table 1. Despite propensity score matching, the following variables remained unbalanced at baseline: meniscal injury, meniscal treatment, cartilage injury, and the KOOS score subcategories of ADL, quality of life (QOL), and symptoms. The ACLR suture augmentation group had a higher incidence of cartilage and meniscal injuries at baseline compared to the control group. The mean difference in KOOS scores was < 10 points and was therefore considered clinically insignificant [6, 20].

**Table 1:** Patient baseline characteristic and operative data.

	InternalBrace (*n* = 162)	Control (*n* = 162)	SMD
Pre‐operative data			
Sex (*n* (%))			0.08
Male	89 (55)	82 (51)	
Female	73 (45)	80 (49)	
Age^a^	24.6 (8.8)	24.5 (8.0)	−0.01
Activity of injury (*n* (%))			
Pivoting	117 (72)	121 (75)	0.06
Non‐pivoting	45 (28)	41 (25)	
Prior surgery (*n* (%))			0.03
No	143 (88)	141 (87)	
Yes	19 (12)	21 (13)	
Side of injury			0.02
Left	80 (49)	82 (51)	
Right	82 (51)	80 (49)	
KT‐1000 (*n* (%)) mm^b^	81 (50) 4.7 (4.2–5.1)	77 (48) 4.8 (4.4–5.3)	0.08
Pivot shift grade, (*n* (%))			0.06
Grade 0	1 (1)	2 (1)	
Grade 1	27 (17)	29 (18)	
Grade 2	88 (54)	102 (63)	
Grade 3	46 (28)	29 (18)	
KOOS scores (*n* (%))	110 (68)	50 (31)	
ADL^a^	83 (16)	86 (15)	0.19
Pain^a^	75 (15)	77 (19)	0.08
QOL^a^	40 (18)	44 (19)	0.22
Sport^a^	46 (26)	47 (26)	0.03
Symptoms^a^	74 (15)	77 (15)	0.19
Operative data			
Cartilage injury (*n* (%))			0.20
No	116 (72)	130 (80)	
Yes	46 (28)	32 (20)	
Meniscus injury (*n* (%))			0.24
No	55 (34)	74 (46)	
Yes	107 (66)	88 (54)	
Meniscus treatment			0.17
No	7 (4)	10 (6)	
Yes	100 (62)	78 (48)	
Missing data	55 (34)	74 (46)	

Abbreviations: ADL, activity of daily life; CI, confidence interval; KOOS, Knee Injury and Osteoarthritis Outcome Score; QOL, quality of life; SD, standard deviation; SMD, standardised mean difference.

^a^ Mean (SD).

^b^ Mean (95% CI).

The median time from injury to surgery was 23 months in the InternalBrace group and 38 months in the control group. The 1‐year follow‐up results are shown in Table 2.

**Table 2:** One‐year follow‐up data and revision rates at 1 and 2 years of follow‐up.

	InternalBrace (*n* = 162)	Control (*n* = 162)	*p* value
KT‐1000^a^	117 (72)	121 (75)	
SSD (mm)^b^	2.2 (1.9–2.4)	1.9 (1.6–2.1)	n.s
Pivot shift^a^	129 (80)	120 (74)	
Negative	100 (62)	76 (47)	0.01^d^
Positive	29 (18)	49 (30)	
Pivot shift by grade^a^			
Negative grade 0	100 (62)	76 (47)	
Positive grade 1	27 (17)	42 (26)	
Positive grade 2	2 (1)	2 (1)	
Positive grade 3	0 (0)	5 (3)	
KOOS^a^	55 (34)	52 (32)	
ADL^c^	97	95	n.s
Pain^c^	89	89	n.s
QOL^c^	63	63	n.s
Sport^c^	75	68	n.s
Symptoms^c^	82	79	n.s
Revision rates^a^			
1 Year	0 (0)	0 (0)	
2 Years	2 (1.8)	3 (1.9)	

Abbreviations: ADL, activity of daily life; CI, confidence interval; KOOS, Knee Injury and Osteoarthritis Outcome Score; QOL, quality of life; SD, standard deviation; SSD, side‐to‐side difference.

^a^ *n* (%).

^b^ Mean (95% CI).

^c^ Median.

^d^ *χ* ^2^ test.

One‐year follow‐up measurements of sagittal knee laxity were available for 117 patients (72%) in the suture augmentation ACLR group and 121 patients (75%) in the control group. The mean sagittal knee laxity was 2.2 mm (95% CI: 1.9–2.4 mm) for the suture augmentation ACLR group and 1.9 mm (95% CI: 1.6–2.1 mm) for the control group. There was no statistically significant difference in sagittal knee laxity between the groups (*p* > 0.5).

One‐year pivot shift testing was available for 129 patients (80%) in the suture augmentation ACLR group and 120 patients (74%) in the control group. In the suture augmentation ACLR group, 29 patients (18%) had a positive pivot shift at 1 year. This compared to 49 patients (30%) in the control group. The suture augmentation ACLR group had a statistically significant lower risk of having a positive pivot shift at 1 year postoperatively compared with the control group (*p* < 0.05).

No difference was observed in any of the KOOS categories between the sutured ACLR and control groups (*p* > 0.05 for all categories). In the InternalBrace group, 55 patients (34%) completed the KOOS questionnaire. In the control group, 52 patients (32%) responded.

At 1 year, there were no revision surgeries in either group. In the ACLR suture augmentation group, 2‐year follow‐up was available for 102 patients, of which two patients (1.8%) had revision surgery. In the control group, 2‐year follow‐up was available for 160 patients, of which three patients (1.9%) had revision surgery (*p* = 0.3).

### Sensitivity analysis

Compared to the matched group (no suture augmentation ACLR), the unmatched group (with suture augmentation ACLR) had older patients (*p* < 0.01), a higher incidence of cartilage injury (*p* < 0.01), fewer injuries related to pivoting (*p* = 0.02), a lower risk of positive pivot shift (*p* = 0.02) and lower scores in the KOOS sports subcategory (*p* = 0.02). There were no differences in the other baseline variables.

A subgroup analysis was performed to compare sagittal knee laxity between the two types of graft used in the suture tape augmentation ACLR group. In the suture tape augmentation ACLR group, 128 patients (79%) received a hamstring tendon autograft, and 34 patients (21%) received a quadriceps tendon graft. Data on postoperative knee laxity were available for 90 patients in the sutured ACLR augmentation group and 27 patients in the control group. The mean sagittal knee laxity was 2.2 mm (95% CI: 1.9–2.5 mm) for the hamstring tendons and 2.0 mm (95% CI: 1.5–2.5 mm) for the quadriceps tendons, with no statistically significant difference (*p* > 0.05).

A subgroup analysis was performed to compare revision rates from the treatment group with the national registry records. After matching, 177 patients with suture‐assisted ACLR from Silkeborg Regional Hospital were matched with 177 patients from the national registry. One patient from the national registry had a revision at the 1‐year follow‐up, while two sutured ACLR patients had a revision at the 2‐year follow‐up. In the suture augmentation ACLR group, there were zero revisions at 1 year and two revisions at 2 years, demonstrating similar revision rates between the groups with no statistical difference in the hazard ratio (*p* = 0.3).

## DISCUSSION

The primary finding of this study was that the ACLR group with suture augmentation did not result in inferior sagittal knee stability. This finding is relevant to the expected benefits of synthetic suture augmentation, where the technique was expected to prevent unnecessary stretching of the ACL graft during rehabilitation. However, no such reduction in knee laxity could be demonstrated at 1 year. The results of the present study are therefore consistent with previous clinical studies that found similar knee laxity between suture‐assisted and conventional ACLR [1, 18, 24]. Similar to the present study these previous studies also did not demonstrate an increase in knee laxity with usage of suture tape augmentation ACLR. An important finding since increased knee laxity has previously been associated with inferior long‐term subjective outcomes [23], suggesting that suture tape augmentation ACLR is safe in terms of knee stability.

A secondary finding was that the suture tape augmentation group had a statistically significant lower degree of positive pivot shift at 1 year follow‐up compared to the control group with 18% and 30% positive pivot shift, respectively. This effect of internal bracing on rotational stability has not previously been demonstrated in previous clinical studies [5, 18, 24], all of which found no difference in postoperative pivot shift. This is an interesting finding in terms of long‐term outcomes and potential impact on degenerative changes after ACLR, as a positive pivot shift is known to be a predictor of early‐onset osteoarthritis [13]. However, it is difficult to explain this finding of reduced positive pivot shift as sagittal stability was not affected by the suture tape augmentation. It may be that the stiffer graft construct, which included a synthetic ligament, had a greater impact on the more complex and combined loading pattern of the pivot shift, while not affecting the more unidirectional sagittal knee stability measure. The proportion of patients with some degree of positive pivot shift at follow‐up was higher than that typically published in ACL reconstruction outcome studies, which are typically in the 10%–15% range. The only possible reason for this could be that the patients included in this study all had independent physiotherapist assessments of knee laxity, which are unbiased compared to the surgeon‐based assessments that are typical in most clinical trials. This more unbiased assessment could result in more patients being classified as having some degree of pivot shift.

There was no difference subjective outcome based on KOOS scores at 1‐year after surgery. This was also the case in the study by Essen et al. [24]. However, the study by Bodendorfer et al. [5] found a significant difference in some of the subcategories of the KOOS score. The study found that QOL improved by 17 points and sport by 12 points [12]. Bodendorfer et al. [5] included allografts, which have previously been associated with improved patient‐reported outcomes, likely due to reduced donor site morbidity load [10].

Another important finding was that the suture augmentation group had similar revision rates to the control group at both 1 and 2 years. These findings are consistent with those of previous trials that have shown equal or lower revision rates in a suture tape augmented group compared to a non‐suture augmentation group [15, 18, 24]. These results suggest that suture tape augmentation did not have an impact on revision rates at 1 and 2 years of follow‐up, which were low in both groups. However, it would be interesting to investigate whether these findings would also be found after a longer follow‐up period or in high‐risk patient groups such as young pivoting athletes. This may be of particular interest as synthetic augmentation may result in stress shielding of the graft and therefore potentially inferior graft maturation, which could lead to a higher risk of subsequent graft failure.

The present study investigated the effect of suture tape augmentation of ACLR in a mixed graft cohort using both hamstring and quadriceps tendon autografts. Allom et al. [1] and Parkes et al. [18] evaluated the effect of suture tape using only hamstring tendon autografts. The choice to combine two types of graft was based on the ability to include the largest possible cohort, thereby strengthening the validity. A subgroup analysis was performed to determine whether there was a difference in knee laxity between the two types of grafts in the suture tape augmentation group to address if the decision of combining two types of graft influenced the results. This was not the case in this study, but in the study by Essen et al. [24], hamstring tendon autografts with suture tape showed reduced knee laxity at six months compared to no suture tape, whereas quadriceps autografts did not.

To the best of our knowledge, this study contains the largest patient cohort to date comparing knee stability, revision rates and clinical outcomes of ACLR with and without suture tape augmentation. By using propensity scoring, this study attempted to reduce the effects of selection bias and confounding based on pre‐existing covariates [14]. By obtaining balanced baseline variables through the matching process, this study improved its internal validity and its ability to assess the effects of internal bracing.

However, some study limitations of the study are worth mentioning. The propensity score method has limitations because it can only adjust for known pre‐existing covariates. Therefore, this retrospective study is still subject to uncontrolled bias. Also, the fact that 92 patients were excluded during the matching process and that they differed from the matched patients in some baseline variables may have led to a possible selection bias This study was constructed with matched cohorts, which strengthens its internal validity; however, the limitation of narrow matching may negatively affect the external validity. Another limitation is the lack of information on patient rehabilitation and return to sport. In Denmark rehabilitation programmes are not standardised; they vary at the municipal level. Another concern could be the that this study used two different time inclusion periods. The inclusion periods were based on when the clinic introduced the use of suture tape augmentation, which could lead to performance bias. However, to reduce the impact of performance bias, a gap of 1 year was used between the inclusion periods to allow surgeons to adapt to the new technique. Finally, this study was limited by the low level of completion of both objective and subjective preoperative data and outcomes from the registry.

By demonstrating better rotational stability, equivalent sagittal stability and equivalent revision rates with the use of a supplemental synthetic ligament added to the ACL graft during ACLR in a large cohort, this study suggests that suture tape augmentation is safe to use in patients undergoing ACLR and that the procedure may have some degree of beneficial postoperative biomechanical knee function. The results of this study therefore add to the existing body of knowledge. In addition, this study is the first to demonstrate a lower risk of a positive pivot shift in a suture tape augmented group compared to a control group. Future research in a large randomised controlled trial would be of interest [11].

## CONCLUSION

Suture augmentation in ACLR reduces the risk of a positive pivot shift at 1 year postoperatively compared to conventional ACLR and demonstrates low rates of revision surgery, similar to ACLR without suture. Suture augmentation also demonstrated similar sagittal knee laxity and PROMs to conventional ACLR. The authors support the use of suture augmentation in ACLR based on the reduction in postoperative pivot shift and the finding that it may reduce sagittal knee laxity.

## AUTHOR CONTRIBUTIONS

**Simone Elmholt**: Project planning; data management and analysis; and article writing. **Torsten Nielsen**: Data extraction; data management; data analysis and article review. **Anders Galaly**: Data analysis and article review. **Mogens Strange**: Surgeon and article review. **Kasper Saxtrup**: Surgeon and article review. **Martin Lind**: Main supervisor; coordinator and article review.

## CONFLICT OF INTEREST STATEMENT

The authors declare no conflicts of interest.

## ETHICS STATEMENT

This study was approved by the Danish Board of Health and the Danish Data Protection Agency (approval no.: 1‐16‐02‐216‐22). Since this study was based on data from a national healthcare registry, approval from the local ethical committee was not necessary.

## Data Availability

With the revised manuscript a zip file with the production data has been added for publication.
